# Extrusion-Based 3D Printing of Photocrosslinkable Chitosan Inks

**DOI:** 10.3390/gels10020126

**Published:** 2024-02-04

**Authors:** Ane García-García, Leyre Pérez-Álvarez, Leire Ruiz-Rubio, Asier Larrea-Sebal, Cesar Martin, José Luis Vilas-Vilela

**Affiliations:** 1 Grupo de Química Macromolecular (LABQUIMAC), Departamento de Química Física, Facultad de Ciencia y Tecnología, Universidad del País Vasco (UPV/EHU), 48940 Leioa, Spain; ane.garcia@bcmaterials.net (A.G.-G.); leire.ruiz@ehu.eus (L.R.-R.); joseluis.vilas@ehu.eus (J.L.V.-V.); 2 BCMaterials, Basque Center for Materials, Applications and Nanostructures, UPV/EHU Science Park, 48940 Leioa, Spain; 3 Biofisika Institute (UPV/EHU, CSIC), UPV/EHU Science Park, 48940 Leioa, Spain; asier.larrea@ehu.eus (A.L.-S.); cesar.martin@ehu.eus (C.M.); 4 Department of Biochemistry and Molecular Biology, Facultad de Ciencia y Tecnología, Universidad del País Vasco UPV/EHU, 48940 Leioa, Spain; 5 Fundación Biofisika Bizkaia, Barrio Sarriena s/n., 48940 Leioa, Spain

**Keywords:** methacrylated chitosan, photocrosslinking, extrusion printing

## Abstract

Photocuring of chitosan has shown great promise in the extrusion-based 3D printing of scaffolds for advanced biomedical and tissue engineering applications. However, the poor mechanical stability of methacrylated chitosan photocuring ink restricts its applicability. The inclusion of co-networks by means of simultaneous polycomplex formation is an effective method by which to solve this drawback, but the formed hydrogel inks are not printable. This work aims to develop new photocurable chitosan inks based on the simultaneous photocrosslinking of methacrylated chitosan (CHIMe) with N,N′-methylenebisacrylamide, polyethylene glycol diacrylate, and acrylic acid to be applied in extrusion 3D printing. Interestingly, the polycomplex co-network corresponding to the acrylic-acid-based ink could be successfully printed by the here-presented simultaneous photocuring strategy. Further, the conversion of photocrosslinking was studied via photo-DSC analyses that revealed a clear dependence on the chemical structure of the employed crosslinking agents (from 40 to ~100%). In addition, the mechanical and rheological properties of the photocured hydrogels were comparatively studied, as well as the printing quality of the extruded scaffolds. The newly developed chitosan photocurable inks demonstrated extrusion printability (squareness ~0.90; uniformity factor ~0.95) and tunable mechanical properties (Young modulus 14–1068 Pa) by means of different crosslinking approaches according to the chemical architecture of the reactive molecules employed. This work shows the great potential of photocrosslinkable chitosan inks.

## 1. Introduction

Tissue engineering and regenerative medicine, which are evolving fields based on the development of new biomaterials, have significantly progressed due to the recent advance of 3D printing techniques. This is based on the fact that tissue development and regeneration strategies require biocompatible 3D scaffolds that act as an extracellular matrix (ECM), providing cells with personalized 3D architectures and serving as a controlled stimuli source for the regulation of cell growth, proliferation, and differentiation [1]. Three-dimensional printing consists of the three-dimensional deposition and sequential layering of materials using a computer-created design. This additive process has opened up new possibilities for designing personalized scaffolds with high precision, reproducibility, and adaptability in comparison with more traditional methods like replica molding.

In the context of an escalating interest in 3D-printable biomaterials, novel ink formulations with selective biological features, high printing fidelity, stability, and appropriate mechanical properties are highly demanded [2].

Among the 3D printing techniques currently used for tissue engineering applications, extrusion-based printing is recognized as a simple additive manufacturing technique that involves the extrusion of cytocompatible materials able to mimic the ECM environment through micrometric nozzles to fabricate three-dimensional structures [3]. Despite its low resolution in comparison with laser-mediated or stereolithographic approaches, extrusion printing, due to its simplicity, is the most-used technique for the 3D printing of polymer hydrogels [3,4].

Hydrogels are hydrophilic polymer networks, recently exploited as inks with which to fabricate scaffolds due to their resemblance to an extracellular matrix (ECM) and their ability to facilitate and regulate cell migration and adhesion [2]. Hydrogel elastomeric networks are formed via the physical or chemical crosslinking of polymer chains with themselves and/or with multifunctional reactive crosslinking agents [5].

In extrusion-based hydrogels printing, as the liquid ink is extruded out of a cartridge, the deposited viscous gel is printed as long as its rheological properties are appropriate for maintaining mechanical stability and shape fidelity [6]. To enable this, together with deposition, a phase transition takes place as a consequence of the physical and/or chemical crosslinking of the polymeric chains [7]. Among the different stimuli that allow for this sol–gel transition of the hydrogel ink [8], it is worth highlighting the widely exploited ionotropic gelation of alginate biopolymer [9,10]. However, light irradiation as the stimuli, and photocuring as a mechanism, have emerged as promising approaches to promoting fast and stable chemical crosslinks, ensuring the higher resolution required for the printing of complex structures.

The most typical photocuring reactions in the biomedical field, together with thiol–ene reactions, are free radical chain reactions [11], which are usually based on vinyl synthetic monomers and oligomers that polymerize through a chain growth mechanism, or vinyl polymers that are chemically crosslinked [12]. Solutions of (meth)acrylate-based monomers/oligomers/polymers are widely employed as 3D photocuring radical inks because they are highly compatible with commercially available 3D printers [13]. Although methacrylates are less reactive than acrylates, they are highly preferred in biomedical applications since they display much lower toxicity [14], which has provoked the recent outbreak of investigations into methacrylated derivatives as photocurable biomaterials [12].

Although a significant advance has been made in the photocuring of hydrogels, as is the case with the successful 3D printing of commercial methacrylated alginate and methacrylated gelatine-based formulations, currently, there is still a real need for a larger variety of printable hydrogel inks to spread out the progress in this field [15].

In the search for new printable inks for tissue engineering purposes, naturally derived hydrogels, that are original materials from an extracellular matrix, biocompatible, biodegradable under physiological conditions, and not toxic, have shown to be excellent candidates [16]. Polysaccharides are specifically interesting as a natural, sustainable, and renewable source of biomaterials, and consequently, current research on natural–hydrogel inks has focused on these biopolymers [10]. Limitations associated with some polysaccharides, such as the high viscosity of agarose or the suboptimal cell attachment and protein absorption characteristics of alginate, have prompted the exploration and utilization of alternative biopolymers.

Chitosan is a naturally derived polysaccharide often used in tissue engineering since, apart from being biocompatible, nontoxic, similar to an extracellular matrix, it degrades to oligomers via lysozyme, which is present in the human body. In addition, chitosan presents unique biological properties, such as its antimicrobial activity, that have driven the growth of chitosan derivatives in all their possible forms [17]. In relation to the applicability of this polysaccharide as a 3D printing bioink, chitosan solutions not only exhibit stability under physiological conditions and suitable viscosity values for bioprinting applications; they are also conducive to proper cell proliferation and differentiation. All this means that chitosan-based inks are positioned at the forefront of candidate inks for 3D bioprinting applications due to their wide viscosity range, diverse crosslinking mechanisms, and adjustable mechanical properties, together with their remarkable cell viability and antibacterial activity [18].

In the context of chitosan crosslinking, it undergoes gelation through both physical and chemical cross-linking mechanisms. However, one notable drawback for tissue engineering applications is the inherently slow gelation rate associated with these mechanisms. This limitation contrasts with the faster process of photocuring. In its original form, chitosan is not inherently photocurable. Nevertheless, recent advancements in the past decade have explored the use of UV light irradiation for chitosan gelation, which is achieved through prior modification via methacrylation reactions. This modified approach presents a more efficient and rapid crosslinking method for chitosan, addressing the time-related challenges associated with traditional gelation mechanisms and enhancing its suitability for applications in tissue engineering. [19]. Methacrylated chitosan has shown the ability to quickly form mechanically stable networks via an in situ photocuring process. Indeed, there are several examples of studies that employ methacrylated chitosan for biomedical purposes [20]. Nevertheless, despite the already-reported investigations on photocrosslinkable chitosan scaffolds [21], 3D printing of stable chitosan-based structures is still a challenging issue that is under early exploration [22].

The main reason behind this is the poor mechanical strength of the singular biopolymer hydrogel. To overcome this drawback, the addition of multifunctional acrylate monomers [14] or polymers [23] acting as covalent crosslinking agents and forming a double-component ink is commonly applied in these cases.

The incorporation of multifunctional monomers acting as crosslinking agents is an interesting alternative because they promote rapid curing and good spatial resolution. However, brittleness and high crosslink density derived from these types of crosslinking agents are serious issues that restrict their applicability, especially in the biomedical field [14].

Among synthetic hydrogels, photocured polyethylene glycol diacrylate (PEGDA) hydrogels have been widely investigated in tissue engineering due to their hydrophilicity, high mechanical stability, biocompatibility, and biodegradability. With this in mind, plenty of examples have arisen in the bibliography that take advantage of the benefits of this synthetic polymer to regulate the mechanical properties, degradation rate, and/or printability of methacrylated natural polymers by preparing chemically formed co-networks by reaction with PEGDA [24,25].

Physical co-networks of photocured chitosan, resulting from additional physical forces such as hydrogen bonding or electrostatic interactions, have also been explored to enhance the mechanical properties of photocrosslinkable methacrylated chitosan [26]. Interestingly, chitosan is able to form polycomplexes with natural or synthetic polyacids in solution via spontaneous association, leading to the formation of strong polycomplex networks without requiring the use of chemical crosslinking agents [27]. The combination of chitosan–polyacid polycomplexes within a photocrosslinked chitosan hydrogel results in doubly interpenetrated networks that have demonstrated tailored stiffness and degradability. However, despite some of these chitosan–polyacid polycomplexes fulfilling the main requirements to be extruded, their high cohesive forces limit their direct printing ability, which has been shown to be restricted to a multimaterial and low-quality layer via the layer printing process [28].

**Figure 1:**
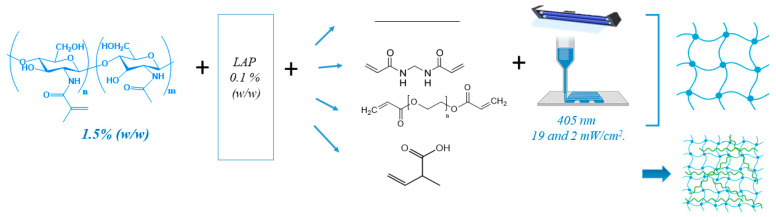
A representative scheme of the synthetic approach for the preparation of methacrylated chitosan hydrogels with NMBA, PEG, and AA as crosslinking agents.

In this context, this work explores the potential photo-induced printing of a new formulation of methacrylated chitosan ink, including acid and photopolymerizable monomeric units that can polymerize during photocuring in combination with chitosan photocrosslinking leading to physical co-networks. This work’s hypothesis is based on the feasibility of the accurate printing of the interpenetrated network of methacrylate chitosan and the polyacid, which is supported by the fact that the polycomplex formation between chitosan and the polyacid takes place after gel deposition and while the photocrosslinking process of the bulk methacrylated network takes place. For this, acrylic acid was chosen as an acidic monomeric precursor for polycomplex formation with methacrylated chitosan. The photoprinting ability and printing quality of acrylic acid-based ink were compared with those of the plain methacrylated chitosan ink and with two different covalently formed co-networks inks. The proposed covalent co-networks were based on the combination of methacrylated chitosan with PEGDA, a multifunctional crosslinking polymer, and N,N′-methylenebisacrylamide (NMBA), a multifunctional and biocompatible crosslinking monomer, which are traditionally employed as crosslinking agent in bulk hydrogels (Figure 1). Moreover, rheological and mechanical properties of photocured hydrogels were also comparatively analyzed.

## 2. Results and Discussion

### 2.1. Hydrogels Photocuring

**Figure 2:**
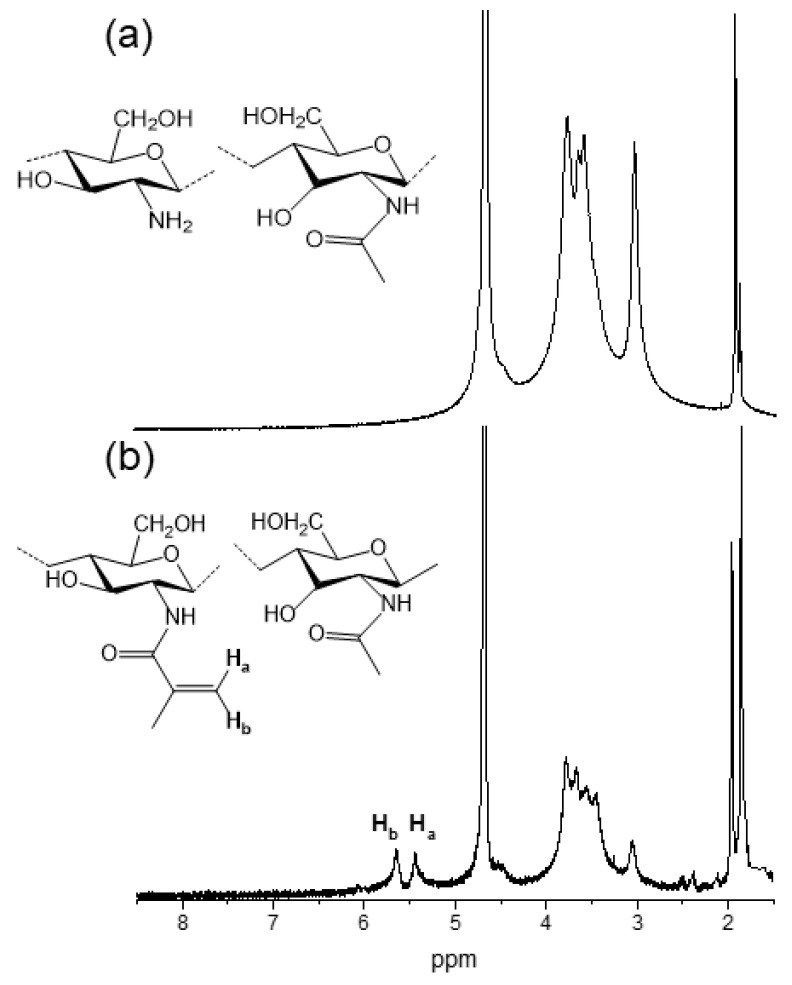
^1^H-NMR spectra of (**a**) pristine chitosan and (**b**) methacrylated chitosan.

Pristine chitosan was modified by reaction with methacrylic anhydride in order to become photocrosslinkable; thus, its solution can act as a photocurable ink after the photoinitiator addition. This modification reaction takes place via the nucleophilic attack of the amine group in chitosan on the carbonyl group in methacrylic anhydride, giving methacrylated chitosan as a product. The successful synthesis was confirmed via ^1^H-NMR spectroscopy (Figure 2).

In the ^1^H-NMR spectra of chitosan (Figure 2a), in addition to the signals of the methyl protons of the acetyl group and the deacetylated proton at 2 and 2.8 ppm, respectively, the signals corresponding to the protons in the glucosamine ring can be seen between 2.8 and 3.9 ppm. In the ^1^H-NMR spectra of methacrylated chitosan (Figure 2b), these same signals are observed, but also those that appeared at 5.6–6.2 ppm, ascribed to the protons of the alkenyl group in the methacrylate moiety. Moreover, at 1.85 ppm, a signal corresponding to the protons in the methyl group of the methacrylate moiety can also be observed [29]. In addition to qualitatively corroborating the success of the modification reaction of chitosan, ^1^H-NMR spectroscopy allowed for the determination of the methacrylation degree. This was calculated via the integration of the protons in the alkenyl group (Ha, Hb) with respect to the integration of the protons in the glucosamine ring, resulting in an average methacrylation degree of 54 ± 9% that enables the further photocrosslinking of chitosan.

LAP photo-initiator was selected due to its high water solubility and visible light-sensitive photoinitiation (405 nm), which leads to high cell viability [30]. Hydrogel inks were prepared by mixing methacrylated chitosan acidic solution with photoinitiator solution and, in the case of AA, NMBA, and PEGDA, co-networks, by adding the corresponding amount of crosslinking agent as described in the Experimental Section.

**Figure 3:**
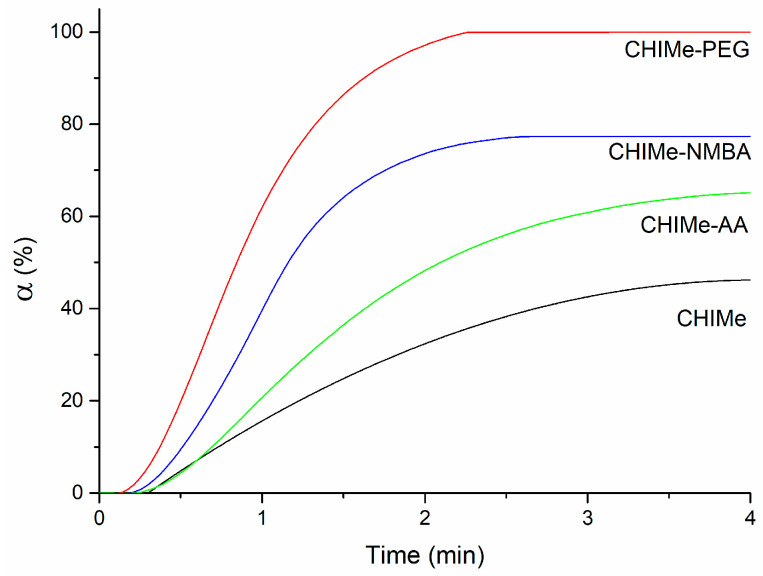
Photo-crosslinking conversion of (black) pristine methacrylated chitosan, and methacrylated chitosan with (red) polyethyleneglycol diacrylate, (blue) N,N′-methylenebisacrylamide, and (green) acrylic acid.

The conversion (*α*) of the photocrosslinking of methacrylated chitosan in the presence of the different vinyl compounds was analyzed via photo-DSC in order to obtain information about the photoprinting ability of the inks (Figure 3).

Regarding the conversion time, it can be observed that all the samples are cured in under four minutes, i.e., times that are short enough to potentially limit the spreading and which favor photocuring-mediated extrusion printing. In comparison with methacrylated chitosan, it can be observed that the incorporation of additional vinyl compounds improves the potential printability of the inks, leading to higher conversion values and lower curing times. The photo-crosslinking study reveals an interesting role of the chemical architecture of the employed vinyl molecules. This could be related to the mobility of the reacting molecules, which, in turn, is closely linked to the final reactivity of the ink. In the absence of external agents, vinyl moieties are present along the chains of high-molecular-weight chitosan chains (highly viscous chitosan), which present reduced mobility; consequently, poor conversion values are registered. However, when smaller bifunctional molecules acting as crosslinking agents, like NMBA and PEGDA, are added, reactivity raises, leading to a great increase in conversion (~2 times) and a reduction in curing time (~2 times). It is worth highlighting the good performance of PEGDA, which can be explained by the well-known flexibility of the linear polyethylene glycol chains. When functionality is taken into consideration, multifunctional monomers show that termination reactions are mobility restricted, resulting in the autoacceleration of the photocuring [14]. The photocuring curve of the ink that presents monofunctional acrylic acid is also in line with this explanation. Accordingly, its monofunctional nature leads to a lower conversion value than when difunctional crosslinkers are employed, for which termination reactions are mobility restricted [14].

### 2.2. Morphology

**Figure 4:**
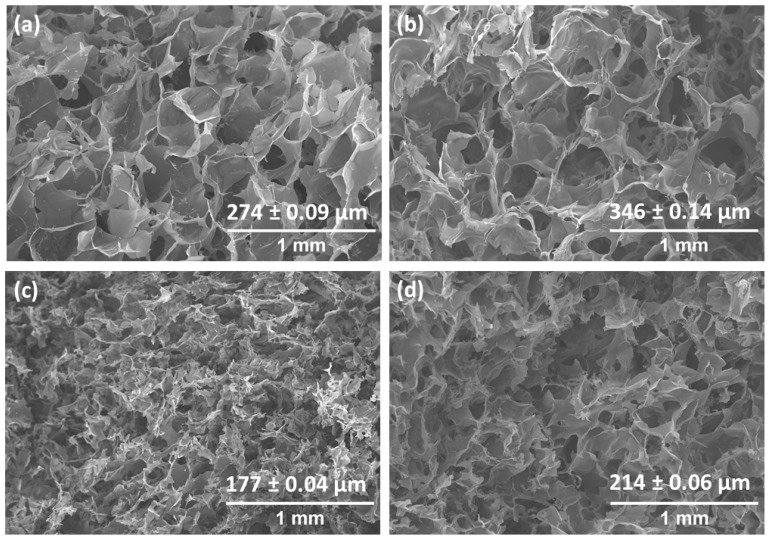
Representative SEM micrographs of CHIMe (**a**), CHIMe-PEGDA (**b**), CHIMe-AA (**c**), and CHIMe-NMBA (**d**) hydrogels.

Figure 4 shows the surface morphology of methacrylated chitosan hydrogels prepared with different crosslinkable agents photographed via scanning electron microscopy (SEM). In all samples, porous and interconnected 3D networks with relatively uniform pore size distributions could be observed, which is of great interest for biological applications. The obtained pore size values were also representative of the molecular structure of the employed vinyl molecules, showing a significant decrease with the addition of acrylic acid and NMBA, while larger pore sizes were measured in the hydrogel crosslinked with PEGDA. According to the results, it must be highlighted that the pore size of PEGDA co-networks seems to be governed by the flexible and hydrophilic structure of PEGDA instead of the higher crosslinking density derived from a higher measured conversion. Thus, the swellable structure resulting from PEGDA-mediated crosslinking favors higher water uptake within hydrogels, leading, after sample freeze drying, to higher pore sizes than in the case of pristine CHIMe network. According to this, rigid and short-length NMBA crosslinking agent promotes smaller pore sizes in the CHIMe-NMBA hydrogels, which is in accordance with average measured value in these samples in comparison with the pore size value of the simple CHIMe hydrogel. Interestingly, as can be observed in Figure 4c, the incorporation of AA in the ink leads to an important decrease in the size of pores. This effect is explained by the formation of the doubly interpenetrated network consequence of the complexation of chitosan with the polyacid. In this sense, the polycomplexation strategy with AA turns out to be a more effective in restricting pore size in chitosan photocrosslinked hydrogels than crosslinking with NMBA.

### 2.3. Rheological and Mechaniccal Properties

**Figure 5:**
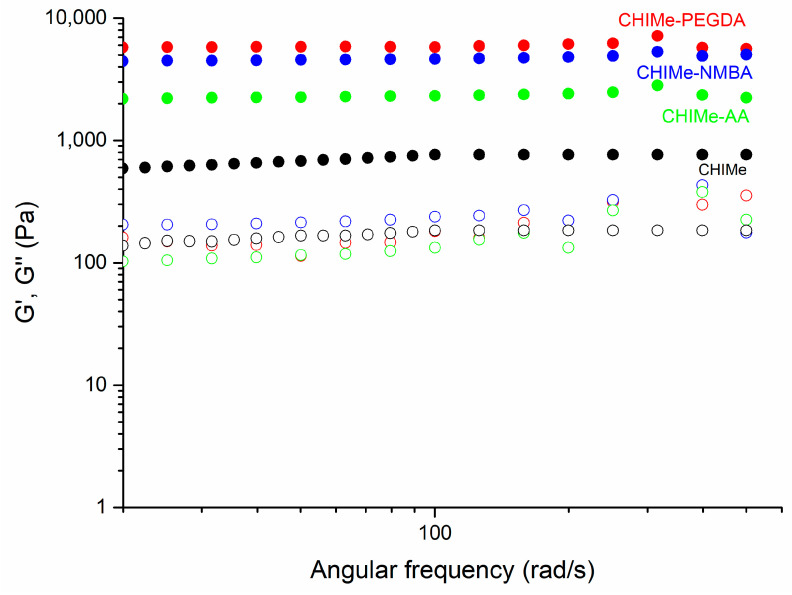
Storage (G′, filled circles) and loss module (G″, open circles) of methacrylated chitosan with NMBA, PEG, and AA as crosslinking agents (1% strain).

The rheological properties of the CHIMe inks prepared with and without the analyzed vinyl compounds were studied by means of frequency sweep measurements. The results obtained in Figure 5 show that all the methacrylated chitosan samples show higher values in storage modulus than in loss modulus (G′ > G″) through the entire frequency range. This behavior means that the elastic properties of the analyzed samples have a greater effect than the viscose ones, which is a typical behavior of stable hydrogels.

It is noteworthy that the incorporation of an external vinyl agent leads to an increase in the storage modulus of the hydrogels (CHIMe-PEGDA, CHIMe-NMBA, CHIMe-AA) with respect to the pristine methacrylated chitosan. Notably, a correlation between the storage moduli of the hydrogels and the photocuring conversion yield determined by photo-DSC measurements can be also observed. Indeed, higher storage moduli correspond to hydrogel samples whichpresent higher photocuring conversion that can be ascribed to a greater crosslinking density and, consequently, to a more solid-like behavior.

**Figure 6:**
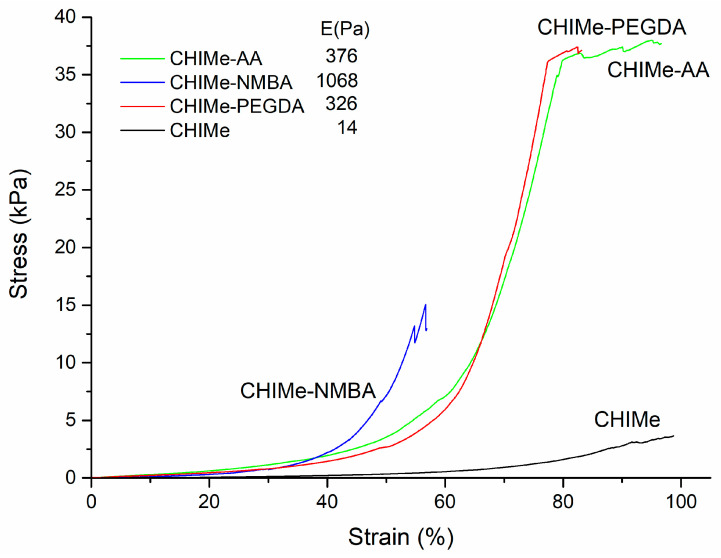
Mechanical stability of methacrylated chitosan with and without crosslinkers under compression stress/strain tests.

Stress–strain compression curves were also obtained for each hydrogel type, as can be seen in Figure 6. In accordance with rheological results, a great improvement in the mechanical properties can be appreciated when external crosslinking agents were added in comparison to CHIMe samples without additional crosslinkers. It is worth highlighting the lower fracture strain shown by the hydrogels crosslinked with NMBA. This brittleness seems to be related to the short length and rigidity characteristic of this crosslinking agent [14]. Indeed, NMBA is typically employed as a successful crosslinker in polycacrylamides hydrogels. These hydrogels are characterized by their own high elasticity, unlike in the case of the here-employed methacrylated chitosan chains. The inherent elasticity of polyacrylamide hydrogels counteracts the rigid nature of the NMBA crosslinking agent, as is observed in methacrylated chitosan networks.

### 2.4. Extrusion 3D Printing

**Figure 7:**
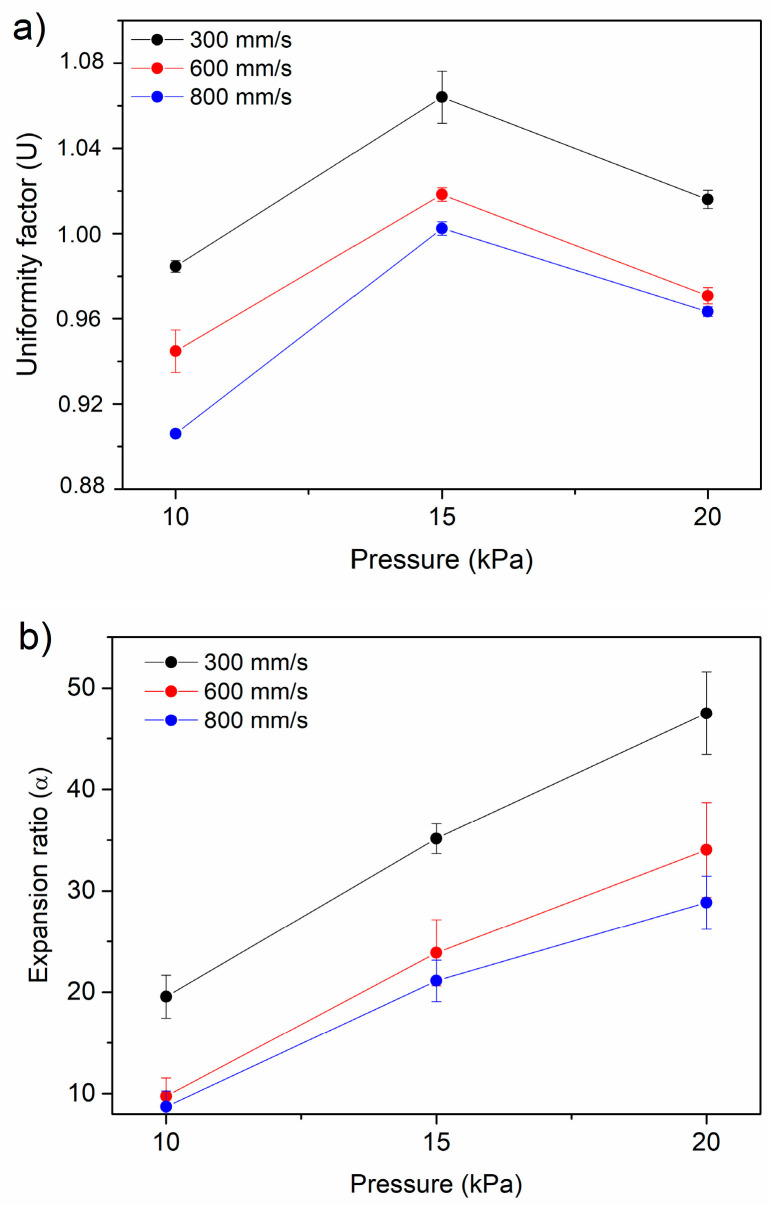
Effect of extrusion pressure (10, 15, and 20 kPa) and printing speed (5, 10, and 13.3 mm/s) on (**a**) uniformity factor, and (**b**) expansion ratio of methacrylated chitosan ink printing using a nozzle diameter of 0.20 mm for methacrylated chitosan.

Nozzle diameter, printing speed and printing pressure were optimized for methacrylated chitosan ink and the results are shown in Figure 7. It can be seen that the expansion ratio decreases with decreasing pressure and increasing speed, while the uniformity factor is closer to 1 for low pressure and decreasing speed. Accordingly, 10 kPa and 10 mm/s were selected as the most accurate pressure and speed printing conditions for all the analyzed methacrylated chitosan-based inks.

**Figure 8:**
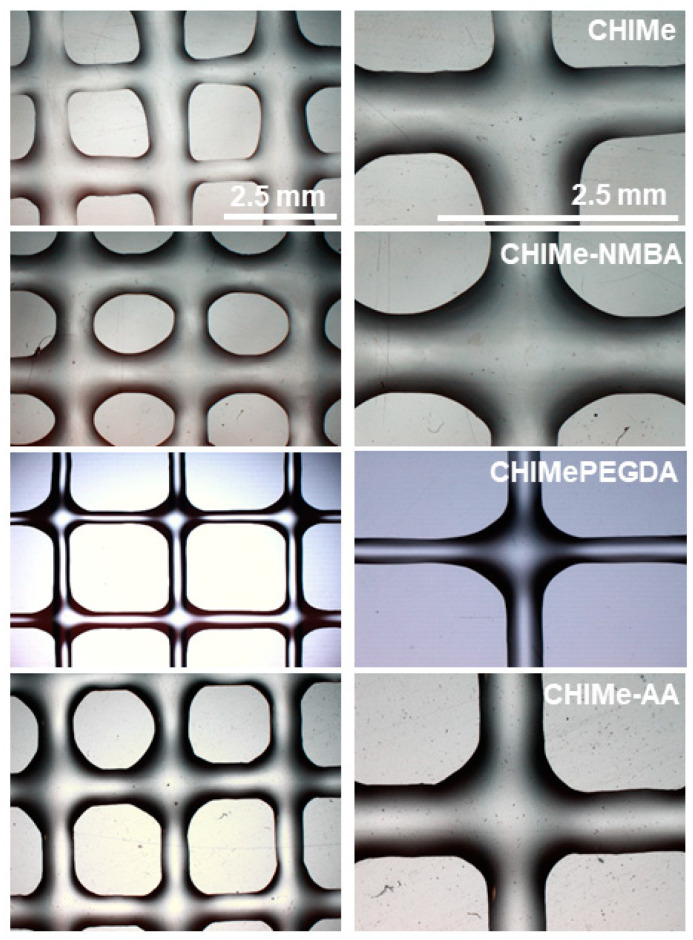
Optical microscope images of 8 × 8 square scaffolds of CHIMe, CHIMe-NMBA, CHIMe-PEG, and CHIMe-AA printed under 405 nm and 190 W/m^2^ light.

With the purpose of studying the effect of the incorporation of selected vinyl compounds on the printability of methacrylated chitosan ink, square-shaped scaffolds were printed (8 × 8 pore per side with an area of 0.25 cm^2^ for each pore) following the above optimized conditions and varying the light intensity (20 and 190 W/m^2^). Optical microscope images of the resulting scaffolds are shown in Figure 8. As can be observed (Figure 8), the ink corresponding to the doubly crosslinked polycomplex (CHIMe-AA) could not only be accurately printed following the same printing conditions as CHIMe pristine ink; it also displayed a higher printing quality.

**Figure 9:**
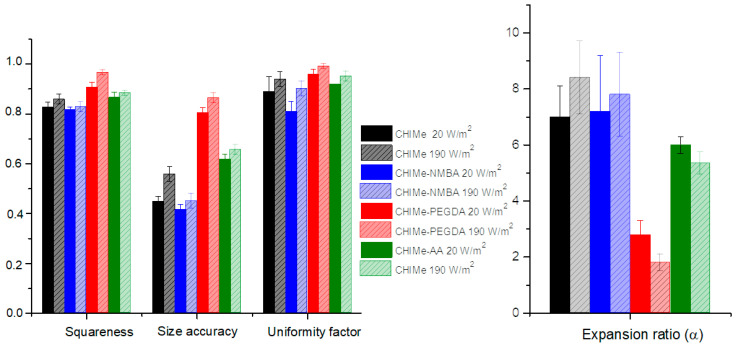
Squareness, size accuracy, uniformity factor, and expansion ratio of 8 × 8 square scaffolds of methacrylated chitosan-derived inks printed under 405 nm light of (filled) 20 W/m^2^ and (patterned) 190 W/m^2^.

According to Figure 9, the addition of external crosslinking in CHIMe ink leads to a clear enhancement of the printability of the inks, increasing squareness, uniformity, and size accuracy while decreasing the expansion ratio, except in the case of CHIMe-NMBA ink. The negative effect on the printability of the addition of this short-length crosslinking agent can be ascribed to the brittleness of photocured CHIMe-NMBA gels, according to the analyses of their mechanical properties. On the contrary, the long-chain and flexible nature of the PEG polymer in the diacrylate crosslinker led to a significantly higher improvement of the printability of the ink attaining squareness and uniformity values close to 1, as well as an important improvement in size accuracy and the expansion ratio. The findings underscore the pivotal role of polymer elasticity in determining printing quality. The observed enhancements in squareness, uniformity, and size accuracy and the reduction in expansion ratio are all indicative of the significant influence that polymer elasticity exerts on the overall printability of the ink. The contrasting outcomes between the short-length crosslinking agent NMBA, leading to brittleness, and the long-chain and flexible PEG polymer, contributing to improved printability, emphasize the importance of selecting crosslinking agents with appropriate elastic properties in 3D printing applications. This insight is valuable for optimizing ink formulations and tailoring them to specific printing requirements, ultimately advancing the quality and performance of the printed structures.

The effect of the light intensity was also tested, observing that, as expected, an increase in light intensity from 20 to 190 W/m^2^ led in all cases to a clear improvement of printing accuracy due to the higher photocuring conversion and thus crosslinking density in the final hydrogels. Although the printing quality of the studied inks seems to be influenced by the type of added crosslinking agent, showing that NMBA derivatives slightly worsened printability, all the inks show favorable printing fidelity. According to this, the mechanical properties of printed hydrogels do not greatly compromise their printability; thus, their stiffness can be tailored varying external crosslinking agent attending only to applicability purposes.

### 2.5. Hydrogel Biocompatibility

**Figure 10:**
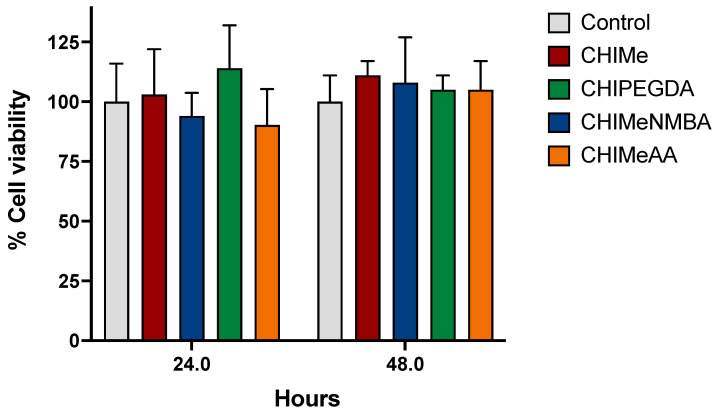
Evaluation of cellular viability in response to hydrogel exposure. HEK293 cells were cultured as indicated in the Materials and Methods section. The cell proliferation assay, assessed via crystal violet staining, reveals comparable viability between cells grown with four distinct hydrogel types and those cultured without hydrogels. Statistical analysis, using the t-Student test, confirms that the observed differences were not statistically significant.

In this study, the potential cytotoxic effects of hydrogels were explored using the cell proliferation assay determined via crystal violet staining. As shown in Figure 10, the cellular viability of cells grown in all four types of hydrogels was comparable to that determined in cells growing in the absence of hydrogels. This observation suggests that the addition of analyzed crosslinking agents did not significantly impact cell viability, indicating the biocompatibility of all the samples under the experimental conditions.

### 2.6. Bactericidal Effects of Hydrogels

**Figure 11:**
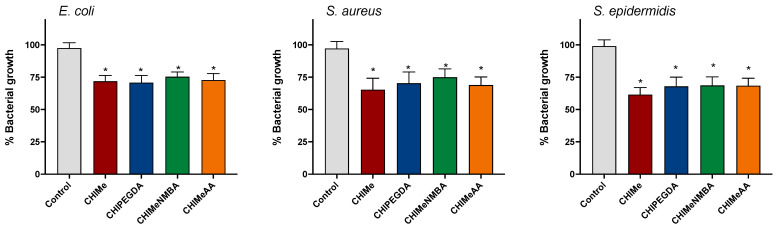
Inhibition of Bacterial Growth by hydrogels. *E. coli*, *S. aureus*, and *S. epidermidis* growth was significantly inhibited after 24 and 48 h of incubation with PET + Curcumin. Error bars represent standard deviations from independent experiments. Statistical significance was determined using Student’s *t*-test (* *p* < 0.001).

The potential bactericidal effects of hydrogels were explored using a bacterial growth assay. As expected, the CHIMe hydrogels resulted in a significant reduction in bacterial growth compared to the control group without hydrogels, observed 24 h post-incubation. As shown in Figure 11, the incorporation of crosslinking agents within CHIMe does not affect observed bacterial growth reduction. This observation underscores the antimicrobial properties of all the hydrogels, implying their potential utility in impeding bacterial proliferation under the experimental conditions.

## 3. Conclusions

New photocurable methacrylated chitosan inks were prepared by the simultaneous photocrosslinking of methacrylated chitosan (CHIMe) with NMBA, PEGDA, and AA. The effect of the addition of AA, PEG, and NMBA as crosslinking agents in the photocuring of methacrylated chitosan ink was evaluated. It was observed that conversion rates and yields of the photocuring were improved with the addition of the analyzed crosslinking agents. Moreover, it could be concluded that conversions were closely related to the mobility and functionality of the employed crosslinking molecule. As expected, all co-networks showed a significant enhancement of the mechanical and rheological properties without a significant detriment to the printing ability, cytocompatibility, and antibacterial properties. Indeed, the polycomplex co-network of CHIMe-AA ink could be accurately printed, unlike traditional chitosan polycomplexes. The chemical structure of crosslinking agents also influences the printability of the gels, and although high resolution was achieved in all the cases, the more-fragile consistency of CHIMe-NMBA hydrogels results in slightly poorer printability. This work shows the great flexibility of photocrosslinkable chitosan as ink for printing with tissue engineering and biomedical purposes.

## 4. Materials and Methods

### 4.1. Materials

For the synthesis of the hydrogels, chitosan from crab shells (Sigma Aldrich, St. Louis, MO, USA, highly viscous) was used, (deacetylation degree of 85% determined by ^1^H-NMR). Acetic acid (for analysis, ≥99.8%) and methacrylic anhydride were also used and, in order to prepare the samples for the NMR study, deuterium oxide (99.9% atom D) and acetic acid-d4 (≥99.5% atom D). Lithium phenyl-2,4,6-trimethylbenzoylphosphinate (LAP) was used as a photoinitiator. Polyethylene glycol (C_2n_H_4n+2_O_n+1_, 700 g/mol) and N,N′-Methylenebisacrylamide (C_7_H_10_N_2_O_2_, 154.17 g/mol) and acrylic acid were purchased from Sigma Aldrich.

### 4.2. Methods

#### 4.2.1. Chitosan Methacrylation

Chitosan was modified via methacrylation reaction with methacrylic anhydride. For this purpose 1.5% (*w*/*w*) 50 mL chitosan solution was prepared in acetic acid 0.5% (*v*/*v*) water solution. Methacrylic anhydride (3.1 mL) was added dropwise to chitosan solution while protecting the mixture from light and left stirring for 24 h at 45 °C. Reaction solution was purified by dialysis against water using 12–14 kDa membranes (5 days) and finally lyophilized (−50 °C and 0.1 mBar).

#### 4.2.2. Preparation of Hydrogels

Previously synthesised methacrylated chitosan (CHIMe) was dissolved (1.5% (*w*/*w*)) in 0.5% (*v*/*v*) acetic acid solution. Separately, LAP photoiniciator (0.1% (*w*/*w*)) was dissolved in 0.5% (*v*/*v*) acetic acid (300 µL) and added to the mixture. Crosslinking agents PEG, NMBA, or acrylic acid (20 mM) were added to the already-prepared CHIMe solution. Photocrosslinking was carried out at 405 nm using UV light LEDs with different intensities (190 and 20 W/m^2^) (Figure 8).

#### 4.2.3. ^1^H-NMR

The average methacrylation degree of chitosan was calculated from ^1^H-NMR spectra. A total of 1.5% (*w*/*w*) methacrylated chitosan solution was prepared in deuterated water, which was acidified with deuterated acetic acid 0.5% (*v*/*v*). ^1^H-NMR spectra were taken on a Bruker Advance 500 MHz spectrometer at 25 °C. In order to determine the methacrylation degree (MD), according to literature [29], the ratio of the relative integrals area of the H2–H6 protons (2.8–3.9 ppm) of N-acetyl-glucosamine units of chitosan was compared to the resonance peaks of methacrylate groups (5.6 and 6.2 ppm) (Equation (1)):

(1) $$MD\left(\%\right)=\frac{\frac{Integral\;of\;methacrylate\;protons\;at\;5.6\;-\;6.2\;ppm}{2}}{\frac{Integral\;of\;chitosan\;cycle\;protons\;at\;2.8\;-\;3.9\;ppm}{6}}\times 100.$$

#### 4.2.4. Photo-DSC Analysis

The UV curing process was studied via photo-DSC using a DSC (TA Instruments Q2000, Waters Corporation, New Castle, DE, USA), which presents a 200 W mercury lamp, an optical range from 320 to 500 nm, and an intensity between 10 and 20 W/m^2^. Conversion degree (*α*) of carbon double bonds was calculated according to Equation (2):

(2) $$m2\alpha =\frac{{∆H}_{t}}{{∆H}^{theor}},$$

where ${∆H}^{theor}$ is the theoretical enthalpy of the fully conversion and ${∆H}_{t}$ is the enthalpy change with time. ${∆H}_{t}$ was obtained by integrating heat flow change, and ${∆H}^{theor}$ was calculated using Equation (3):

(3) $${∆H}^{theor}=\frac{54.7\times Funtionality}{M},$$

where $M^{theor}$ is the molecular weight of chitosan, 54.7 kJ/mol is the molar enthalpy of the methacrylate group [31], and functionality is 1 in the present case.

#### 4.2.5. Scanning Electron Microscopy (SEM)

The morphology and the pore size of the prepared hydrogels were analyzed with a Hitachi S-3400N scanning electron microscope (SEM), (150 s, 20 mA, 15 kV, ×50,000 amplification). Previously, different chitosan hydrogels were lyophilized (−50 °C, 0.1 mBar) and coated with a thin gold overlay before SEM characterization. Pore size was determined using ImageJ 1.49 software. At least 40 pores were analyzed in each sample. The error of the distribution is represented as the mean ± standard deviation.

#### 4.2.6. Rheology

The rheological behaviors of chitosan-based photocrosslinkable hydrogels were analyzed in an Advanced Rheometric Expansion System (ARES TA instruments with peltier oven (APS), Waters Corporation, New Castle, PA, USA) using parallel plate geometry (25 mm of diameter) with a gap distance of 1.7 mm. The effect of increasing angular frequency on the samples was measured in order to evaluate the storage (G′) and loss modulus (G″). Angular frequencies from 0.1 to 500 rad/s at a constant strain of 1% were used for the measurements.

#### 4.2.7. Mechanical Properties

The mechanical properties of the prepared hydrogels were analyzed with material-testing equipment using a 20 N load cell (Metrotec, MTE-1, Lezo, Spain). For this purpose, compression stress–strain curves were obtained following a deformation speed of 1 mm/min. Young’s moduli were calculated in the range of 45–60% strain (%).

#### 4.2.8. Printing Parameters

Printing parameters were optimized for methacrylated chitosan ink using an INKREDIBLE+ Cellink bioprinter (Cellink, Gothenburg, Sweeden). To this purpose, straight lines were printed using cartridges (Adhesive Dispensing Ltd., Bletchley, UK) with a nozzle diameter of 0.20 mm, varying printing speed (5, 10 and 13.3 mm/s) and extrusion pressure (10, 15 and 20 kPa).

The 8 × 8 pores scaffolds (0.25 cm^2^ pore) of methacrylated chitosan-based inks were printed using the 0.20 mm nozzle at 10 mm/s and 10 KPa. Printability was analyzed via the processing (ImageJ 1.49 software) of scaffolds imagages with the following parameters.

The expansion ratio (Equation (4)) that represents the relation between the diameter of the printed filament (d) and the theoretical diameter of the nozzle (D):

expansion ratio (*α*) = d/D. (4)

The uniformity factor (Equation (5)), that is, the ratio between the printed (l) and the theoretical (L) length of the filament:

uniformity factor (U) = l/L.(5)

Size accuracy according to (Equation (6)) [32], in which A_t_ is the theoretical area of the pore and A is that of printed squares:

(6) $$size\;accuracy=1-\frac{A_{t}-A}{A_{t}}.$$

Squareness was calculated according to (Equation (7)) [33], where L is the perimeter of the pore and A is the following area:

(7) $$Squareness=\frac{L^{2}}{16A}.$$

#### 4.2.9. Crystal Violet Assay for Determining Cytotoxicity

HEK293 cells (40 × 10^3^) in DMEM supplemented with 10% FBS (*v*/*v*), 100 µg/mL streptomycin, 100 U/mL penicillin, and L-glutamine were seeded in 96-well culture plates containing the different hydrogels and allowed to grow for 24 and 48 h. The cells were washed with phosphate-buffered saline (PBS) and fixed with 4.5% paraformaldehyde in PBS for 30 min at room temperature. After fixation, cells were washed with PBS again and stained with 0.5% solution of crystal violet for 20 min at room temperature. Cells were then washed thoroughly in water. After washing, 200 µL of acetic acid (15%) was added to each well, and the plate was incubated for 20 min at room temperature on a bench rocker with a frequency of 20 oscillations per minute. Finally, acetic acid was transferred to a new 96-well culture plate, and the optical density (OD) of each well was measured at 570 nm (OD570) with a plate reader. The percentage of viable cells was calculated by comparing the average OD570 values of cells seeded directly in the 96-well culture plate with the OD570 values of the cells seeded in the hydrogels.

#### 4.2.10. Determination of Bacterial Growth by Spectrophotometry and Viable CFU/mL

*Escherichia coli* (ATCC25922), *Staphylococcus aureus* (ATCC29213), and *Staphylococcus epidermidis* (ATCC35984) were used as test microorganism in this study. All the strains were stored as frozen stocks with 15% glycerol at −80 °C. The bacteria were cultured in Luria Bertani (LB) broth or LB agar.

Overnight bacterial cultures from single colonies grown on agar plates were diluted 10-fold in fresh medium and incubated at 37 °C until the turbidity reached an optical density between 0.08 and 0.100 (1.5 × 10^8^ CFU/mL) corresponding to 0.5 McFarland, measured via spectrophotometry at 600 nm. The inoculum (200 μL) containing 5 × 10^5^ CFU/mL of each strain was added to wells of microtiter plate containing the different hydrogels measuring, the turbidity at 600 nm in a plate Reader (Bio-Tek Instruments, Santa Clara, CA, USA). The effect of the hydrogels’ presence on bacterial growth was determined by the percentage reduction in bacterial growth compared to the bacterial growth in the presence of PET.

## Data Availability

All data and materials are available on request from the corresponding author. The data are not publicly available due to ongoing research using part of the data.
